# Machine Learning Allows for Distinguishing Precancerous and Cancerous Human Epithelial Cervical Cells Using High-Resolution AFM Imaging of Adhesion Maps

**DOI:** 10.3390/cells12212536

**Published:** 2023-10-28

**Authors:** Mikhail Petrov, Igor Sokolov

**Affiliations:** 1 Department of Mechanical Engineering, Tufts University, Medford, MA 02155, USA; mikhail.petrov@tufts.edu; 2 Departments of Physics and Biomedical Engineering, Tufts University, Medford, MA 02155, USA

**Keywords:** cancer detection, machine learning, atomic force microscopy (AFM), scanning probe microscopy (SPM)

## Abstract

Previously, the analysis of atomic force microscopy (AFM) images allowed us to distinguish normal from cancerous/precancerous human epithelial cervical cells using only the fractal dimension parameter. High-resolution maps of adhesion between the AFM probe and the cell surface were used in that study. However, the separation of cancerous and precancerous cells was rather poor (the area under the curve (AUC) was only 0.79, whereas the accuracy, sensitivity, and specificity were 74%, 58%, and 84%, respectively). At the same time, the separation between premalignant and malignant cells is the most significant from a clinical point of view. Here, we show that the introduction of machine learning methods for the analysis of adhesion maps allows us to distinguish precancerous and cancerous cervical cells with rather good precision (AUC, accuracy, sensitivity, and specificity are 0.93, 83%, 92%, and 78%, respectively). Substantial improvement in sensitivity is significant because of the unmet need in clinical practice to improve the screening of cervical cancer (a relatively low specificity can be compensated by combining this approach with other currently existing screening methods). The random forest decision tree algorithm was utilized in this study. The analysis was carried out using the data of six precancerous primary cell lines and six cancerous primary cell lines, each derived from different humans. The robustness of the classification was verified using K-fold cross-validation (K = 500). The results are statistically significant at *p* < 0.0001. Statistical significance was determined using the random shuffle method as a control.

## 1. Introduction

Morbidity and mortality associated with cervical cancer are substantially decreased when cancer is detected early [1,2,3]. Thus, the search for new approaches to early diagnosis is of great significance. Cervical cancer is the second most frequent type of cancer among women worldwide, with approximately 288,000 deaths per year; more than 14,000 women were diagnosed with this disease in 2022 in the US alone [4,5]. The mortality rate is second only to that for breast cancer. The early detection of cervical cancer using the Papanicolaou (Pap) smear test has decreased mortality from cervical cancer by 70–80% [4,6]. Early detection is based on the identification of neoplastic cells in stained preparations obtained from the uterine cervix [2,6].

It should be noted that, currently, recently introduced DNA-HPV tests are broadly used. It is a good screening test because approximately 95% of cases of this cancer correlate with the presence of human papillomavirus (HPV). However, the accuracy of cancer detection (sensitivity) in DNA-HPV tests is low. For example, only 43% of HPV-positive females diagnosed in Massachusetts (USA) from 2004 to 2014 had cervical cancer [7]. Recent studies have shown that DNA-HPV tests are particularly ineffective among young women who have this virus much more frequently than cervical cancer. Thus, the interest in Pap smear tests is growing again.

The main advantages of Pap smear cytological tests are their simplicity and minimal invasiveness (the cells are obtained from the cervix using a combination of a spatula and brush). Despite the impressive success of these tests, their sensitivity for detecting preinvasive cervical lesions is far from desirable, with a mean sensitivity of only 47% (range 30–80%). The sensitivity of the cytological tests for invasive carcinoma (cancer cells) is not perfect, ranging from 16% to 82% in different studies [4,8]. According to the American Cancer Society data, each year, in the United States alone, approximately 3.6 million cytological tests are classified as equivocal, out of which only 8% of women will have preinvasive (high-grade squamous intraepithelial) lesions, and 0.4% will have carcinoma as found in further testing that involves invasive tissue biopsies. This means that more than 3.3 million biopsies per year may be unnecessary if a more accurate screening of cervical cancer is developed. In addition to the relatively low sensitivity of cytological tests and the moderate reproducibility of the diagnosis, there are sampling and laboratory errors. Finally, there are inherent problems in the identification of malignant cells with cytological test methodology. More accurate tests may substantially decrease the cost and patient inconvenience of screening by eliminating additional steps of colposcopy [9]. However, further improvement in cytological tests may have fundamental restrictions inherent to the limitations of morphologic analysis by means of optical microscopy.

Here, we used a method for the analysis of the cervical epithelial cells by means of atomic force microscopy (AFM). This type of microscopy has a substantial advantage in terms of resolution (200–5000 times) compared with optical microscopy. It is superior to even electron microscopy when applied to soft materials. There have been multiple partially successful attempts to use the surface analysis of cell images to identify cancer cells [10,11]. The AFM technique has been previously used to study cells [12,13,14,15], including cancerous cervical cells [16,17]. Recently introduced AFM modalities such as peak force QNM, HarmoniX, ringing mode, etc., allow for the determination of different physical properties of the cell surface, which is impossible to obtain with any other microscopy method. 

Previously, we have developed a method for the analysis of the AFM-collected maps of adhesion between the AFM probe and the surface of human cervical epithelial cells [18,19]. The cells needed for this approach can be collected using methods similar to Pap smear cytological tests [19,20]. Essentially, the difference is in the use of different fixative, temperature, and time of fixation. The analysis has shown that it is possible to unambiguously distinguish normal from cancerous/precancerous cells by just using one parameter of the fractal dimension of the adhesion maps. Being definitely innovative, the practical utility of such a finding was limited because the separation between precancerous and cancerous cells was rather poor. Specifically, the area under the curve (AUC) was only 0.79, whereas the accuracy, sensitivity, and specificity were 0.74, 0.58, and 0.84, respectively. From a clinical utility point of view, the separation between premalignant and malignant cells would be the most significant because it leads to improvement in the existing Pap smear tests. It is rather difficult to distinguish between such cells using only optical microscopy. 

Here, we show that the introduction of machine learning methods for the analysis of adhesion maps leads to a substantial improvement in the detection of cancer cells with respect to the previous results based on only the fractal dimension. (The values of AUC, accuracy, sensitivity, and specificity are 0.93, 83%, 92%, and 78%, respectively.) It has to be stressed that the increase observed in sensitivity while keeping the number of missed cancers (false negatives) low is the currently existing unmet need in clinical practice. With a sensitivity rate of 92%, the percentage of missed cancer cases (false negative rate) is only 7%, which is much better compared with the 27% achieved with modern invasive colposcopy tests [21]. The analysis is applied to the adhesion maps collected on six primary precancerous and six primary cancerous cell lines; each cell line was extracted from a different patient. 

## 2. Results and Discussion

**Figure 1:**
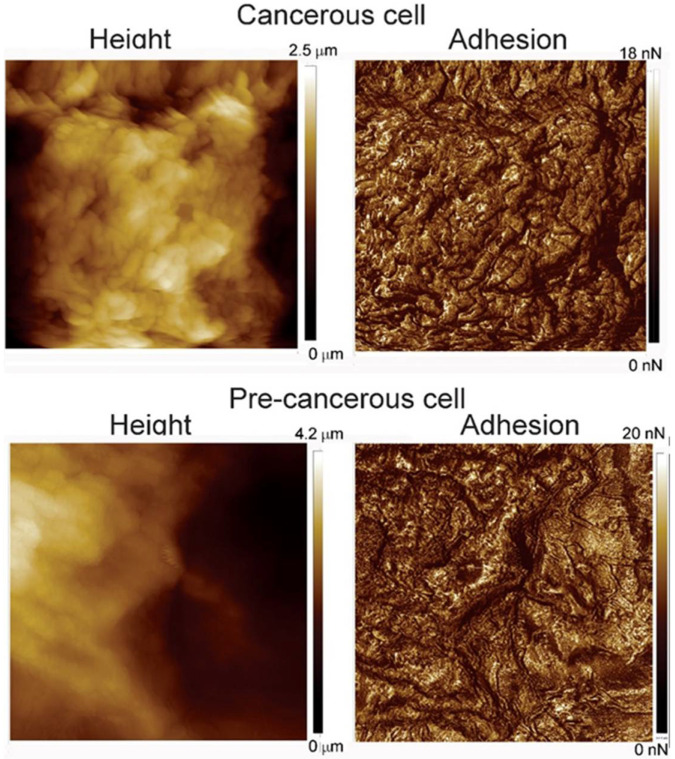
Examples of typical 10 × 10 µm^2^ AFM images of precancerous and cancerous cells used in this study.

As has been found in previous studies [18,19], 10 × 10 µm^2^ is the most effective AFM scan size to discriminate between normal and cancerous cells. Figure 1 shows examples of typical height and adhesion images of this size for cancerous and precancerous cells. The main conclusion from these images is the difficulty of discriminating between these cell phenotypes by just visual judgment. To discriminate between normal and cancerous cells in a quantitative way, it was suggested to investigate the characteristics of the fractal geometry of the cell surface and their adhesion maps [18,19]. The same studies showed that the height images provide very little discriminating power compared with the adhesion maps. Following this conclusion, we will not use the height images to analyze the differences between cancerous and precancerous cells and focus on the adhesion images.

Currently, the most popular image recognition is deep learning, specifically convolution neural networks (CNNs) (see, e.g., [22,23]). However, this analysis works well if there is a sufficient number of images available for training. The AFM technique, due to being a relatively slow microscopy method, does not allow for the generation of a very large number of images. This leads to a number of challenges in building CNN algorithms when applied to the classification of cells [24]. Furthermore, neural networks are particularly prone to overtraining [25,26]. As a result, we suggest using other machine learning algorithms, like decision trees. Specifically, we used a bootstrap of decision trees called random forest [27,28,29]. The important part of our approach is the reduction in data space. Instead of analyzing 512 × 512 pixels to characterize each AFM scan, we converted each scan into a set of “surface parameters”. These parameters are used in engineering to describe the quality of surfaces; the description of these parameters is provided in in detail in ref. [30]. We chose three surface parameters in addition to the three fractal dimension parameters introduced in [18,19]. These parameters were chosen because they demonstrated the greatest difference in their average values between cancer and normal cells. These parameters (their detailed description is provided in the Methods section) represent various characteristics of the surface roughness. 

**Figure 2:**
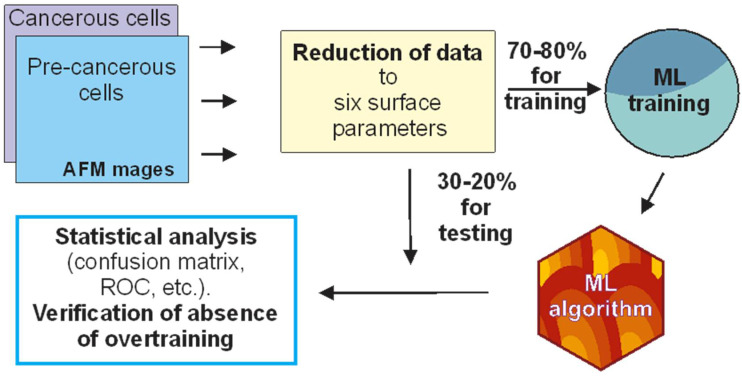
Schematics of machine learning (ML) analysis. Conversion of the AFM images into the surface parameters; splitting the database into the training and testing subsets; developing an ML algorithm using just the training subset; using the testing subset to perform the statistical analysis of the developed ML algorithm; and finally, cross-validation and the verification of the lack of overtraining of the developed approach.

Figure 2 demonstrates the entire process of data analysis. All AFM images of the adhesion were converted into a set of the chosen six surface parameters. Then, the obtained database was randomly split into the training and testing subsets. The split was chosen to be 70% and 80% for the training and 30% and 20% for the testing subsets, respectively. The final result was the same for these two particular splits. After creating the machine learning algorithm by training random forest on the training subset, the statistical analysis of the obtained algorithm was performed on the testing subset. The statistical analysis includes the ROC (receiver operating characteristic) curve and the confusion matrix. ROC curve allows us to define a range of sensitivity (“accuracy” in the identification of cancer cells), specificity (“accuracy” in the identification of the absence of cancer cells), and accuracy. These quantities are defined as follows: sensitivity = TP/(TP + FN); specificity = TN/(TN + FP); accuracy= (TN + TP)/(TP + FN + TN + FP). In these equations, TP, TN, FP, and FN are the true-positive, true-negative, false-positive, and false-negative components of the confusion matrix. 

Note that the ROC curve presumably provides the most comprehensive information about the performance of a particular classifier. It shows the relationship between sensitivity and specificity for a range of thresholds that define the boundary between cancerous and precancerous cells. A classifier used to build the ROC curve gives the probability that a particular cell is cancerous. However, to define if this cell is cancerous or not, one needs to introduce a threshold above which the cell is considered cancerous. The ROC curve is the plot of sensitivity (also called true positive rate) versus specificity (more precisely, 1-specificity, also called the false-positive rate) for the entire range of thresholds ranging from 0% to 100%.

Because the machine learning algorithms are too complicated to be easily verified, it is important to test the robustness of the obtained results. This is carried out by using the K-fold cross-validation and verification of the absence of overtraining. To perform the K-fold cross-validation, we repeated the random split between testing and training databases 500 times (we also observed that a further increase in the number of these splits to 1000 times did not change the obtained statistics). In this way, we can calculate not only the average values of the ROC curve and the components of the confusion matrix but also their standard deviations. Technically, this approach verifies the absence of overtraining because the calculated statistics are determined using the testing subsets, which are completely separated from and independent of the training ones. For example, if the distribution of the area under the ROC curve (AUC) is rather narrow (a small standard deviation compared with the average), the algorithmic approach has no overtraining.

**Figure 3:**
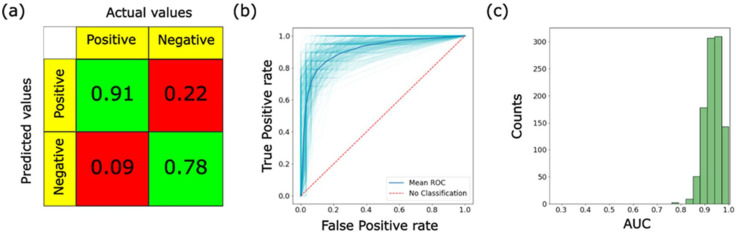
Results of the ML analysis of the difference between precancerous and cancerous cells: (**a**) confusion matrix, (**b**) ROC curves, and (**c**) histogram of the areas under the curve (AUC).

Figure 3 shows the results of the described analysis. The confusion matrix demonstrates the average values of TP, TN FP, and FN equal to 0.91, 0.78, 0.22, and 0.09, respectively. AUC, accuracy, sensitivity, and specificity are 0.93, 83%, 92%, and 78%, respectively. This is a substantial improvement compared with the previously reported values of 0.79, 74%, 58%, and 84%, respectively. It is important to note that, at first glance, this improvement might not look that substantial because of a slight decrease in the specificity. However, it is the increase in sensitivity that is urgently needed in clinical practice. As we described in the Introduction, the existing screening tests for cervical cancer (Pap smear) are effective (decreased mortality from cervical cancer by 70–80%) but insufficiently accurate (mean sensitivity of only 47% (range 30–80%)). This leads to a high number of unnecessary invasive biopsies (colposcopy). Other broadly used screening HPV tests demonstrate 95–100% specificity; however, they have low sensitivity (HPV tests do not detect cancer but only identify people at high risk of getting this cancer). As was mentioned, only 43% of HPV-associated cancers diagnosed in Massachusetts females from 2004 to 2014 had cervical cancer. As a result of the low sensitivity of the existing noninvasive tests, millions of invasive and expensive biopsies (colposcopies) are performed each year. If there was a noninvasive screening test with 95% sensitivity, 3.1 million biopsies per year in the US (based on 2022 data) could be unnecessary. Therefore, the fact that we observed a substantial improvement in sensitivity (58% → 92%) is very promising.

It also should be noted that the most accurate measure of improvement in the method is the value of AUC. The other statistical parameters discussed here depend on a particular threshold of the probability of the classifier, which defines to which class each tested cell belongs. Moving this threshold, one can, for example, increase sensitivity at the expense of specificity. To avoid this ambiguity, one should remember the final goal of the described method, i.e., the detection of the presence of cancer cells. Secondly, the method should not miss cancer cases to the maximum possible extent. The latter is described by the false-negative rate, which is calculated as FN/(FN + TP). For the calculations presented in this work, the false-negative rate is within 7%. This is substantially better than the modern invasive colposcopy tests [21], which miss cancer cases at 27%. It can be improved even further by combining several cells for diagnosis. Nevertheless, it should be borne in mind that the present results were obtained using cell lines. So, it is proof of the concept. To claim the development of a new clinical method, the present method has to be applied to the cells obtained from actual patients.

**Figure 4:**
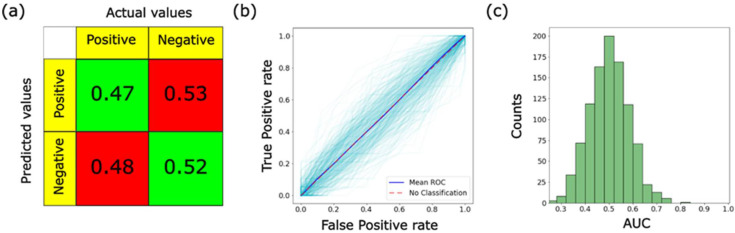
Further verification of the lack of overtraining of the ML algorithm used in this work; shuffled class assignment of the testing dataset: (**a**) confusion matrix, (**b**) ROC curves, and (**c**) histogram of the areas under the curve (AUC). These AUC data were also used to find the statistical significance of the results shown in Figure 3.

Although the random forest method is known not to have serious problems with overtraining, it is instructional to further verify the absence of overtraining. To this end, we used the method suggested in [31]. Furthermore, this method allowed us to find the statistical significance of the obtained results. In this method, it was suggested that a correct algorithm without overtraining should give the correct AUC of 0.5 (no-classification value) for a completely randomized class assignment of the test dataset (the training datasets used to train the algorithm stay the same). The same should be observed for the confusion matrix, which should result in the accuracy, sensitivity, and specificity equal to 50%. To exclude a coincidental choice of the correct class assignment, the process of randomization was repeated 500 times as well (the same idea as in the K-fold cross-validation). Figure 4 demonstrates the results of such calculations. The area under the ROC curve is indeed equal to 0.5, with rather high precision. The average accuracy is also around 50%.

The described above data obtained on the completely randomized test sets can also be used to find the statistical significance of the results of this work. For this, we suggest comparing the AUC data obtained on the actual dataset and the AUC data obtained using the same algorithm (built on the actual training dataset) but applied to the fully randomized test sets. Since the method correctly identified the absence of any signal (randomness of the class association), it can be considered as a control dataset. Using the one-way ANOVA statistical test and the data generated for 500-fold cross-validation, we found that the obtained results are statistically significant at the confidence level *p* < 0.0001.

Let us now discuss the nature of the observed differences between precancerous and cancerous cells. The difference in the pericellular layer of cancerous and normal cells has been reported in the literature [32] using atomic force microscopy, in particular, the modality capable of imaging the distribution of the adhesion. This is novel because it gives unique information about the cell surface, which includes not only the surface morphology of cells but also the maps of the adhesive properties of cells. Additionally, because it is novel, there is no biochemical information available that connects such properties with known biochemical pathways. The previously observed difference in the cell microvilli of cancerous cells [17] is unlikely to be the only reason responsible for the observed difference because such structures are detectable using electronic and even optical microscopy, which has been broadly used in multiple reports in which no substantial difference between normal or cancer cells was found. In addition, the topographical AFM images of the cell surface, in which the microvilli and microridges were perfectly seen, did not show a statistically significant difference between cancer and normal cells [18,19]. This might be because AFM maps demonstrate much higher resolution than topographical images. The AFM lateral resolution is mainly defined by the area of contact between the AFM probe and the sample surface. The increase in resolution in the case of adhesion imaging results from the physics of adhesion measurement. The adhesion is equal to the force experienced by the AFM probe at the moment of disconnection from the sample surface. Because our sample is cells, the pulling of the probe from the cell surface leads to the formation of a neck, the area of which is much smaller than the area of contact between the AFM probe and cell surface at the moment of reaching the load force. The latter is when the topographical image is recorded.

It is instructive to note the possibility of using functionalized AFM probes, which would be sensitive to particular molecules on the cell surface. While it could provide interesting and novel information about the distribution of particular molecules over the cell surface, it is unlikely to provide information that could be used for AFM image analysis using machine learning. This is because functionalized AFM probes are prone to rapid degradation. At the same time, it is paramount for machine learning to have a database of images obtained in a highly repeatable way. Therefore, our choice in the described method was a regular nonfunctionalized AFM probe composed of silicon (which is oxidized in air to silica). The next note is about the use of fixed cells and imaging in air. The method of fixation used here was specifically developed to preserve the cell surface. Secondly, this makes the approach more suitable for clinical applications because it is more practical in the clinical environment to deal with stable fixed cells. Imaging in air provides a higher resolution than imaging in an aqueous environment. In an aqueous environment, there is interference between the AFM probe and the long polysaccharide molecules covering the cell surface. In many cases, it even does not allow for the development of any adhesion between the AFM probe and the cell surface.

The complexity of the challenge to link the observed data to the biochemical processes inside the cell was demonstrated in ref. [19] via the analysis of fractal dimensions, in particular, the concept of multi-practicality. Fractals are complex disorderly patterns that typically occur under far-from-equilibrium conditions [33] and/or emerge from chaos [34]. Fractal shapes are found in the large-scale structure of the universe [35], continental coastlines [36], trees [37], grain structures of materials [38], clouds [39], and even artistic creations [40]. There are many models describing the emergence of fractal geometry [34,41]. However, none of the models explains the emergence of fractal geometry on cells. Therefore, we expect this to be a challenge for future research. Besides being a fundamentally different level of knowledge about the cancer process, the suggested method can be directly used for practical medical applications like the detection of cervical cancer, specifically in combination with the existing screening HPV and Pap smear tests. The test based on the described method can seamlessly be implemented in the clinical practice by utilizing the liquid cytology version of Pap smear tests, in which cells from each patient are available for further fixation and AFM examination. 

In conclusion, it is worth discussing the applicability of the described method to other cancers. A somewhat similar method was successfully applied to detect the presence of bladder cancer using cells extracted from patients’ urine [31]. Using two cell lines of colorectal cancer of different aggressiveness, we demonstrated that AFM imaging can successfully identify the type of cell, with an accuracy of 94% at the level of a single cell [42]. Thus, we can speculate that the described method can be applied to multiple cancers.

## 3. Methods

### 3.1. Cells and AFM imaging

The cell lines, AFM imaging, and sample preparation were described in our previous works [18,19]. Here, we only briefly describe it. Twelve different human cervical epithelial cell lines were prepared from tissues collected from the transformation zone of the cervix from six cancer and six healthy patients. The human tissues were received from the Cooperative Human Tissue Network. Cells were extracted via a two-stage enzymatic digestion with a dispase enzyme to remove the epithelium and then trypsin to disperse the individual epithelial cells [43]. To prepare precancerous (immortal) cells, normal cells (extracted from healthy individuals) were transfected and then immortalized using the HPV-16 virus. After a number of passages, all the cells not immortalized died out after 60–150 population doublings. All cells were used for experiments when cells were subconfluent (<50% confluency). Created this way, precancerous CX-16–2, CX-16–4, CX-16–11, CX-16–12, CX-16–14, and CX-16–15, as well as cancerous CXT-2, CXT-3, CXT-5, CXT-6, CXT-7, and CXT-8 cell lines were used for AFM imaging. In total, images of 64 cancerous cells and 108 precancerous cells were used in this study. The data used in this work can be downloaded from the online database [44].

For the AMF study, cells were fixed with Karnovsky’s fixative as described in [18,19]. In brief, cells cultured in 60 mm Petri dishes were washed twice with PBS buffer and then treated with 4 ml of Karnovsky’s fixative overnight at 4 °C. The fixed cells were flushed from Karnovsky’s fixative with 5 mL of DI water twice (one time for several hours). For imaging, cells were dried using a freeze dryer and stored in a desiccator. As we showed in ref. [19], cells should be imaged under humidity not exceeding 60–65%. The actual imaging was carried out under the relative humidity not exceeding 50%. While the actual sizes of the measured cells ranged between 20 and 40 µm, a 10 × 10 µm^2^ scanning area was chosen at random for each cell to avoid any possible operator bias.

A Nanoscope™ Dimension 3100 AFM (Veeco/Bruker-Nano, Inc., Santa Barbara, CA, USA) with Nanoscope V controller working in HarmoniX mode was employed. A standard cantilever holder with HarmoniX standard cantilevers for operation in air was used. The HarmoniX mode is a variant of subresonance tapping, in which the force–distance curve is restored at each tap [45]. The maximum pull-off force required to disconnect the AFM probe from the sample is recorded as the force of adhesion. While there are other AFM modes with which the force of adhesion can be recorded, such as force–volume and other subresonance tapping modes, HarmoniX is noticeably faster.

### 3.2. Surface Parameters Used in This Study

One AFM 10 × 10 µm^2^ adhesion map was recorded per cell at 512 × 512 pixel resolution. The map was then divided into four 5 × 5 µm^2^ quadrants at 256 × 256 pixel resolution each. The surface parameters were determined for each quadrant. If the average and median values of the four quadrants differed by more than 50%, the cell map was visually verified for possible artifacts (a piece of dirt on the cell surface or picking dirt using the AFM probe). If an artifact was identified in a quadrant, the quadrant was removed from consideration. The surface parameters were averaged for the remaining quadrants per cell.

The surface parameters used in this work include surface area ratio (*Sdr*), root mean square gradient (*Sdq*), reduced summit height (*Spk*), and the fractal dimension. Besides these, we also used two fractal dimensions introduced in our previous work [18,19], *Sfd_top*, and *Sfd_bottom*, which are the fractal dimensions calculated for the surface features above and below 300 nm in size, respectively. Below, we provide the formulas for the calculation of these surface parameters. 

*The surface area ratio (Sdr)* represents the increase in the interfacial surface area relative to the area of the projected (flat) *x*, *y* plane:

$$S_{\;sdr}=\frac{\left(\sum_{k=0}^{M-2}\sum_{l=0}^{N-2}A_{kl}\right)-\left(M-1\right)\left(N-1\right)\delta x\delta y}{\left(M-1\right)\left(N-1\right)\delta x\delta y}100\%,$$

where *A_kl_* is defined as

$$\begin{array}{l} A_{kl}=\frac{1}{4}\left(\sqrt{\delta y^{2}+{\left(z\left(x_{k},y_{l}\right)-z\left(x_{k},y_{l+1}\right)\right)}^{2}}+\sqrt{\delta y^{2}+{\left(z\left(x_{k+1},y_{l}\right)-z\left(x_{k+1},y_{l+1}\right)\right)}^{2}}\right)\cdot \\ \left(\sqrt{\delta y^{2}+{\left(z\left(x_{k},y_{l}\right)-z\left(x_{k+1},y_{l}\right)\right)}^{2}}+\sqrt{\delta y^{2}+{\left(z\left(x_{k},y_{l+1}\right)-z\left(x_{k+1},y_{l+1}\right)\right)}^{2}}\right) \end{array}$$

*The root mean square gradient (Sdq)* is the RMS value of the surface slope within the sampling area. It is defined as

$$S_{dq}=\sqrt{\frac{1}{\left(M-1\right)\left(N-1\right)}\sum_{k=0}^{M-1}\sum_{l=0}^{N-1}{\left(\frac{z\left(x_{k},y_{l}\right)-z\left(x_{k-1},y_{l}\right)}{\delta x}\right)}^{2}+{\left(\frac{z\left(x_{k},y_{l}\right)-z\left(x_{k},y_{l-1}\right)}{\delta y}\right)}^{2}}.$$

*Reduced summit height (Spk)* is calculated using an algorithm in an implicit way. It is calculated based on the bearing area ratio curve. To find this parameter, a line needs to be drawn, which is fitted to the 40% segment of the curve that results in the lowest decline (using the least mean squares). Then, this line is extrapolated until it crosses the vertical axes of the bearing area ratio curve for 0% and 100%. Two horizontal lines are drawn through the intersection points. Finally, a straight line that starts at the intersection point between the bearing area ratio curve and the upper horizontal line and ends on the 0% axis should be drawn in a way that the area of the obtained triangle is the same as the area between the horizontal line and the bearing area ratio curve. Using the same algorithm, a line between the lower horizontal line and the 100% axis should be drawn. The reduced summit height (*Spk*) is the value of the height of the upper left triangle.

*Fractal dimensions* were calculated with the help of SPIP software following the method described in [41]. The method is based on the use of two-dimensional Fourier transformation:

$$F\left(u,v\right)=\frac{1}{N_{x}N_{y}}\sum_{x=0}^{N_{x}-1}\sum_{y=0}^{N_{y}-1}z\left(x,y\right)e^{-i2\pi \left(ux/N_{x}+vy/N_{y}\right)},$$

where *N_x_* and *N_y_* are the number of pixels in the *x* and *y* directions, and *u* and *v are* the discrete Fourier indexes =0, 1, 2, … *N_x_*_−1_ and *v* = 0, 1, 2 … *N_y_*_−1_.

The obtained Fourier spectrum was averaged over all possible directions to convert this spectrum into 1D. The resulting spectrum is only a function *A(Q)* of reciprocal space coordinate *Q* or the inverse lateral size of the geometrical features on the AFM image. The fractal dimension was calculated using the expected power-law behavior *A(Q) ~ Q^b^*. Specifically, the fractal dimension was defined as *2−b.* Two fractal dimensions were calculated, below (*Sfd_top*) and above (*Sfd_bottom*) Q = 1/300 nm^−1^. Both fractal dimensions were used in the machine learning analysis described in this work as two separate parameters.

It should be noted that, ideologically, the use of the surface parameters to analyze the maps of adhesion is a substantial departure from the classically considered surface parameters. When we use the previously suggested formulas, they contain a mix of quantities of different dimensions. Although it does not imply any specific restrictions when these parameters are used in machine learning, obviously, the answer should depend on which particular units we use. For example, many parameters will be different if we use the presentation of adhesion force in N (Newtons), µN, or nN. Therefore, the choice of particular units has to be consistent along all measurements. Specifically, here, the largest difference between cancerous and precancerous cells was observed when the adhesion force was used in nanoNewtons, and the spatial dimensions were in the nanometers.

## Data Availability

The data that support the findings of this study are available upon a reasonable request from the corresponding author.
